# Fatal Hemophagocytic Lymphohistiocytosis Associated with Locally Acquired Dengue Virus Infection — New Mexico and Texas, 2012

**Published:** 2014-01-24

**Authors:** Tyler M. Sharp, Linda Gaul, Atis Muehlenbachs, Elizabeth Hunsperger, Julu Bhatnagar, Rebekka Lueptow, Gilberto A. Santiago, Jorge L. Muñoz-Jordan, Dianna M. Blau, Paul Ettestad, Jack D. Bissett, Suzanne C. Ledet, Sherif R. Zaki, Kay M. Tomashek

**Affiliations:** 1 Division of Vector-Borne Diseases, National Center for Emerging and Zoonotic Infectious Diseases, CDC; 2 Texas Department of State Health Services; 3 Division of High Consequence Pathogens and Pathology, National Center for Emerging and Zoonotic Infectious Diseases, CDC; 4 New Mexico Department of Health; 5 Seton Medical Center Austin, Texas

Dengue is caused by infection with any of four mosquito-transmitted dengue viruses (DENV-1–4) and is characterized by fever, headache, myalgia, and leukopenia (1). Hemophagocytic lymphohistiocytosis (HLH) is a potentially fatal hyperinflammatory syndrome that can be familial or acquired, and is characterized by persistent fever, pancytopenia, hepatosplenomegaly, and increased serum ferritin (2). Acquired HLH is most frequently associated with Epstein Barr virus infection but also has been associated with dengue (3). This report describes a fatal case of acquired HLH that was apparently triggered by infection with DENV-3. The patient developed an acute febrile illness in August 2012 during a 1-month vacation in New Mexico. After returning to her home in Texas, she was initially diagnosed with West Nile virus (WNV) infection, developed pancytopenia, liver failure, and disseminated intravascular coagulopathy, and died. DENV-3 was detected in a premortem bone marrow biopsy in which erythrophagocytosis was evident. This case underscores the need for clinicians in the United States to be vigilant for dengue and request diagnostic testing for suspected cases, which should be reported to public health authorities.

## Case Investigation

**FIGURE 1:**
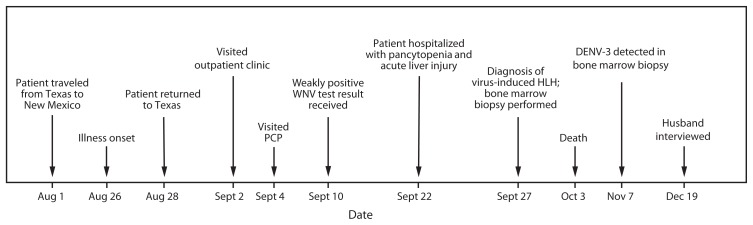
Timeline of events surrounding the illness of a woman with fatal dengue-associated hemophagocytic lymphohistiocytosis — New Mexico and Texas, 2012 **Abbreviations:** PCP = primary care physician; WNV = West Nile virus; HLH = hemophagocytic lymphohistiocytosis; DENV-3 = dengue virus-type 3.

**FIGURE 2:**
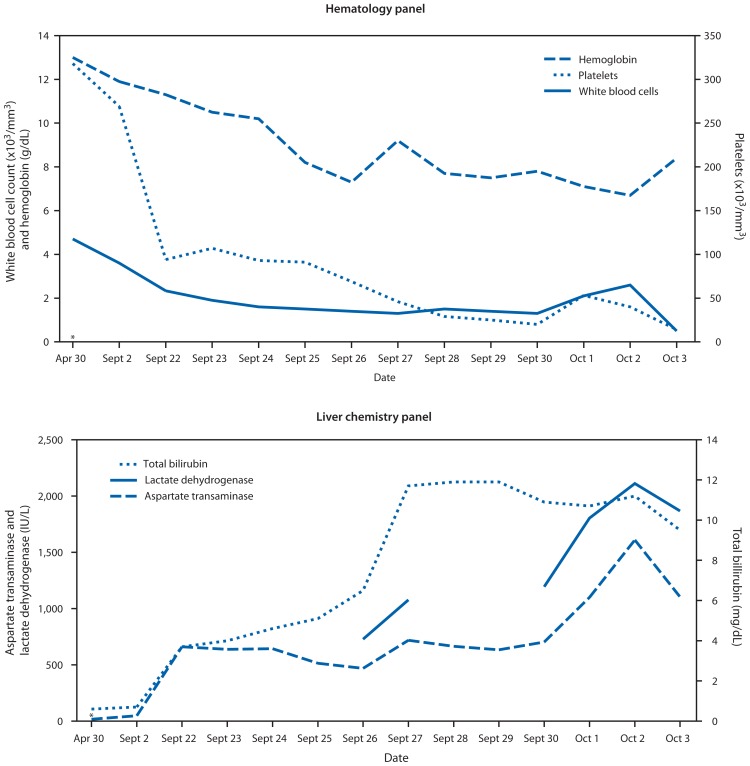
Key laboratory values from hematology and liver chemistry panels for a woman with fatal dengue-associated hemophagocytic lymphohistiocytosis — New Mexico and Texas, 2012 * Laboratory values collected during routine physical examination, before illness onset.

On September 2, 2012, a woman aged 63 years went to an outpatient clinic in central Texas with a 7-day history of fatigue, anorexia, headache, hematuria, and leg pain (Figure 1). She had a history of Crohn’s disease treated with mercaptopurine and mesalamine, hysterectomy because of uterine cancer, thyroidectomy, hypertension treated with lisinopril, coronary artery disease, hyperlipidemia, chronic renal disease with microhematuria, obesity, and depression treated with fluoxetine. Upon examination, the patient was febrile, hypotensive, and had low oxygen saturation (SaO_2_ = 92% [normal = ≥95%]). Laboratory values revealed leukopenia (white blood cell count = 3,600/mm^3^ [normal = 3,800–10,600/mm^3^]) (Figure 2, top panel). She was diagnosed with dehydration, given 1 L of intravenous normal saline, and instructed to see her primary care physician if her symptoms did not resolve.

On September 4, the patient went to her primary care physician and reported fatigue, anorexia, headache, leg cramps, fever, and chills. She did not have respiratory, gastrointestinal, or urinary symptoms. Physical examination revealed hypotension and fever. Lisinopril was discontinued, serum was drawn for typhus and WNV serology, and doxycycline was prescribed. A weakly positive anti-WNV immunoglobulin M (IgM) diagnostic test result was received on September 10, and the patient was prescribed bed rest for 2 weeks. Doxycycline was discontinued because of negative typhus serology.

On September 22, the woman sought care at a regional emergency department because of persistent fatigue, fever, and chills. At triage, the patient was hypotensive, tachycardic, afebrile, and had low oxygen saturation (SaO_2_ = 90%). Icteric sclerae were noted on physical examination. Laboratory results revealed thrombocytopenia (platelet count = 94,000/mm^3^ [normal = 140,000–400,000/mm^3^]) and anemia (hemoglobin = 11.3 g/dL [normal = 12.0–16.0 g/dL]) (Figure 2, top panel). Results also revealed acute liver injury (aspartate aminotransferase = 662 IU/L [normal = 10–42 IU/L]); total bilirubin = 6.5 mg/dL [normal 0.2–1.2 mg/dL]) (Figure 2, bottom panel). She was transferred to a tertiary care hospital for inpatient management. At admission, the patient reported extreme fatigue, difficulty walking, shortness of breath without cough, anorexia, fever, chills, and dark urine. She continued to be hypotensive and was given 1 L of intravenous normal saline. Mercaptopurine and mesalamine were discontinued. Diagnostic testing was negative for infection with hepatitis A, B, and C viruses.

On hospital day 2, the patient continued to be afebrile, hypotensive, and tachycardic with no adenopathy or organomegaly. Abdominal ultrasound revealed diffuse fatty infiltration of the liver. Tests to detect antinuclear antibodies were negative. On hospital day 3 she developed bibasilar posterior respiratory crackles and was transferred to the intensive care unit for placement of a central venous catheter. On hospital day 4 she developed pitting pedal edema, wheezing with productive cough, and bleeding from the site of central venous catheter insertion. Serum ferritin was increased (>7,500 ng/mL [normal range = 7–282 ng/mL]) as was partial thromboplastin time (>250 seconds [normal range = 25.1–36.5 seconds]). Serum fibrinogen was decreased (<60 mg/dL [normal range = 200–393 mg/dL]). On September 26 (hospital day 5), the patient had fever, tachypenia, neutropenia (absolute neutrophil count = 900/mm^3^ [normal = 1,500–8,000/mm^3^]), and elevated liver enzymes (lactate dehydrogenase = 727 IU/L [normal = 91–180 IU/L]). Chest radiography revealed infiltrates with an opacity in the lower lobe of the left lung. Titers taken to detect antinuclear antibodies and rheumatoid factor were positive, a presumptive diagnosis of virus-induced HLH was made, and a bone marrow biopsy and aspiration was performed.

Over the next 7 days, the patient developed bilateral pleural effusions, splenomegaly, anasarca, hemoptysis, and watery diarrhea with blood. A core needle liver biopsy on September 27 (hospital day 6) revealed fulminant hepatitis suggestive of a viral process with no overt hepatocyte necrosis. Serum specimens were collected on hospital day 7 for *Rickettsia* and repeat WNV serology, which were negative and weakly positive, respectively. Although the result of reverse transcription–polymerase chain reaction (RT-PCR) testing to detect WNV nucleic acid was negative on the same day, interferon therapy was initiated because of the possibility of WNV-induced hepatitis. Hemodialysis was initiated on hospital day 10 because of kidney failure, and the following day the patient developed respiratory distress and was intubated.

The patient received a diagnosis of severe metabolic acidosis and volume overload on hospital day 11, and soon after became encephalopathic and unresponsive. Palliative care was initiated, and the previously collected liver and bone marrow biopsies were sent to CDC for confirmation of WNV infection. On October 3, the patient died. She had been administered a total of 27 units of blood products during hospital days 4–12 because of bleeding. Retrospective medical record review confirmed that the case met the HLH clinical case definition (2).

**FIGURE 3:**
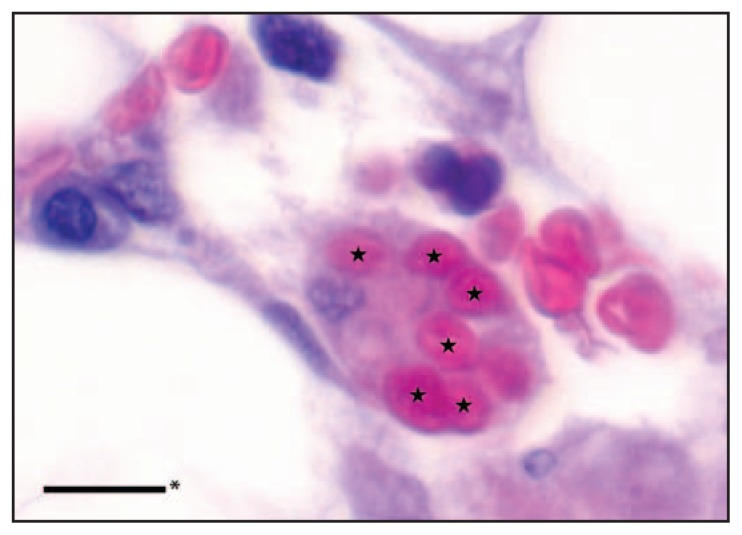
Stars indicate erythrocytes phagocytized by a macrophage in a hematoxylin and eosin–stained section of bone marrow biopsy from a patient with fatal dengue-associated hemophagocytic lymphohistiocytosis — New Mexico and Texas, 2012 * Scale bar represents 10 micrometers.

The liver biopsy showed extensive liver damage, including marked steatosis and ballooning degeneration with neutrophilic and histiocytic aggregates and portal lymphocytic infiltrates. Macrophages with intracellular erythrocytes were noted in the bone marrow biopsy (Figure 3). RNA was extracted from the bone marrow aspirate and tested by RT-PCR assays specific for WNV and flaviviruses; the assay results were negative and positive, respectively. Sequencing of the flavivirus-specific PCR product on November 7 revealed 98% nucleotide identity with DENV-3, which was confirmed with DENV-type specific RT-PCR. Anti-DENV immunohistochemistry was negative in both the liver and bone marrow biopsies. Special stains for acid-fast bacilli, fungi, and bacteria and immunohistochemistry for *Coxiella burnetti* on the liver biopsy were negative. No premortem or postmortem blood specimens were available for diagnostic testing.

## Epidemiologic Investigation

Interview of the patient’s husband on December 19 revealed that the vacationing couple had traveled from Texas on August 1 to Santa Fe, New Mexico, where they went for regular walks and frequently spent evenings on their patio. The patient and her husband visited an international fair featuring American Indian arts and crafts during August 18–19. In part because of the patient’s illness, the couple returned to Texas on August 28. The patient had not been outside the continental United States since May 2012, when she visited France. She had previously visited several locations in the tropics, and lived in Bermuda for 1 year in the early 1970s.

Four other persons traveled to Santa Fe with the couple, none of whom reported fever in the 2 weeks before or after the patient’s illness onset. All five of her travel companions provided a serum specimen for detection of anti-DENV IgM and IgG antibodies, and none had evidence of recent or past DENV infection, respectively.

Eighteen persons from Texas donated blood that was given to the patient before the bone marrow biopsy was performed on September 27. Of 17 donors who were contacted, none reported fever in the 2 weeks before or 1 week after donating blood. Fourteen donors provided a serum specimen for diagnostic testing, and none had evidence of recent or past DENV infection.

### Editorial Note

This is the third locally acquired dengue-related death documented in the 50 United States; all three occurred in the past 10 years and were geographically associated with Texas (4,5). Dengue is endemic throughout the tropics, and recent estimates suggest that 390 million DENV infections occurred worldwide in 2010 (6). The majority of dengue cases reported in the 50 United States are travel associated (7). However, because approximately 80% of all laboratory-positive dengue cases tested at private laboratories during 2008–2011 were not reported to public health authorities (CDC Dengue Branch, unpublished data, 2014), the actual incidence of dengue in the United States is unknown.

HLH was recently estimated to have a prevalence of 1 per 100,000 children in Texas and a survival rate of 67% (8). Familial HLH typically manifests early in life and is invariably fatal without treatment, including chemotherapy and immunotherapy followed by hematopoietic stem cell transplantation (2). HLH in adolescents and adults is more often acquired following infection or malignancy and can be successfully treated with therapy against the trigger and corticosteroids (2). Crohn’s disease and immunosuppression are associated with an increased risk for developing HLH (2,9) and might have contributed to development of HLH in the patient described in this report. Also consistent with HLH, the patient had elevated transaminases, bilirubin, and lactate dehydrogenase (2) and all the known risk factors for death in adult HLH patients, including age >30 years, jaundice, disseminated intravascular coagulopathy, and absence of lymphadenopathy (10). HLH is a rare complication of dengue (3), with only 27 cases documented since 1966, including eight (30%) fatal cases. Clinicians in areas with endemic dengue should be aware of dengue-associated HLH because the clinical similarity of severe dengue and HLH might contribute to underrecognition of HLH.

Approximately 95% of persons with dengue will experience an acute febrile illness without clinically significant hemorrhage or plasma leakage (1). Because of nonspecific signs and symptoms, such cases can be misdiagnosed as influenza, WNV infection, or another common acute febrile illness. Although the patient described in this report initially received a diagnosis of WNV infection because of a weakly positive serologic test result, the result likely was produced by crossreactive anti-DENV IgM antibody. Clinicians should be aware of this possible crossreaction when evaluating patients with suspected WNV infection, especially those with recent travel to the tropics. Physicians and public health professionals in the United States should be vigilant for dengue, particularly in the context of ongoing WNV outbreaks and where competent DENV vectors (e.g., *Aedes aegypti* and *Aedes albopictus* mosquitos) are present.

What is already known on this topic?

Dengue is a potentially fatal acute febrile illness caused by infection with any of four mosquito-transmitted dengue viruses and is endemic throughout the tropics; most reported cases in the 50 United States are in travelers. Hemophagocytic lymphohistiocytosis (HLH) is a potentially fatal clinical syndrome that can be acquired following dengue virus infection.

What is added by this report?

The case described in this report represents the third locally acquired dengue-related death in the 50 United States and the first dengue-associated HLH case documented in the country.

What are the implications for public health practice?

Clinicians and public health professionals in the United States should be vigilant for and report cases of travel-associated and locally acquired dengue and request that both molecular and serologic diagnostics be performed in suspected cases. Clinicians in areas with endemic dengue should be aware of HLH as a potential complication of dengue and of the recommended HLH treatment regimen.

Although the location where the patient became infected with DENV-3 could not be conclusively identified, there are several possible scenarios. The DENV incubation period ranges from 3 to 10 days (1), and the patient was in Santa Fe for 26 days (August 1–26) before illness onset. Although competent mosquito vectors of DENV are not known to establish stable populations at elevations above approximately 5,577 feet (1,700 meters) (1), and Santa Fe sits at an elevation of 7,260 feet (2,213 meters), an imported mosquito might have survived in the warmer August climate, fed on a DENV-infected person, and subsequently infected the patient. Alternatively, infection via contaminated blood products is a rare route of DENV transmission, and this route of transmission could not be ruled out because four blood donors did not provide a serum specimen for testing. Finally, it is possible that the patient’s initial illness was caused by an unidentified agent, and she was infected with DENV-3 while en route to or in Texas, after which she developed HLH caused by infection with the unidentified agent and/or DENV-3.

Clinicians in the United States should be aware of dengue and request diagnostic testing that includes both molecular and serologic diagnostics for patients with dengue-like symptoms. Competent DENV vectors are present in most states, and importation of DENV via travelers has resulted in recent dengue outbreaks in Florida, Hawaii, and Texas. All suspected dengue cases should be reported to state and local health departments.

Persons living in or traveling to areas where a risk for dengue exists should avoid mosquito bites by using insect repellent, staying in residences with air conditioning or intact mosquito screens on windows and doors, and emptying or covering all water containers that can serve as mosquito breeding sites.* A world map showing locations of current reports of dengue activity is available online from CDC.†

## Footnotes

* Additional information available at http://www.cdc.gov/dengue/prevention.

† Available at http://www.cdc.gov/dengue.
